# Patient perceived barriers to exercise and their clinical associations in difficult asthma

**DOI:** 10.1186/s40733-020-00058-6

**Published:** 2020-06-09

**Authors:** Anna T. Freeman, David Hill, Colin Newell, Helen Moyses, Adnan Azim, Deborah Knight, Laura Presland, Matthew Harvey, Hans Michael Haitchi, Alastair Watson, Karl J. Staples, Ramesh J. Kurukulaaratchy, Tom M. A. Wilkinson

**Affiliations:** 1 Clinical & Experimental Sciences, University of Southampton Faculty of Medicine, Southampton General Hospital, Southampton, UK; 2 Wessex Investigational Sciences Hub, University of Southampton Faculty of Medicine, Southampton General Hospital, Southampton, UK; 3 Southampton NIHR Respiratory Biomedical Research Centre, Southampton General Hospital, Southampton, UK; 4 Asthma, Allergy and Clinical Immunology Department, University Hospital Southampton NHS Foundation Trust, Southampton, UK; 5 Institute for Life Sciences, University of Southampton, Southampton, UK; 6 The David Hide Asthma & Allergy Research Centre, St Mary’s Hospital, Newport, Isle of Wight UK

**Keywords:** Asthma, exercise, barriers, psychology

## Abstract

**Background:**

Exercise is recommended in guidelines for asthma management and has beneficial effects on symptom control, inflammation and lung function in patients with sub-optimally controlled asthma. Despite this, physical activity levels in patients with difficult asthma are often impaired. Understanding the barriers to exercise in people with difficult asthma is crucial for increasing their activity, and in implementing successful, disease modifying, and holistic approaches to improve their health.

**Methods:**

62 Patients within the WATCH Difficult Asthma Cohort (Southampton, UK) completed an Exercise Therapy Burden Questionnaire (ETBQ). The results were analyzed with contemporaneous asthma-related data to determine relationships between perceived exercise barriers and asthma and comorbidity characteristics

**Results:**

Patients were reflective of a difficult asthma cohort, 66% were female, and 63% were atopic. They had a high BMI (median [inter-quartile range]) of 29.3 [25.5–36.2], age of 53.5 [38.75, 65.25], impaired spirometry with FEV1 73% predicted [59.5, 86.6%] and FEV/FVC ratio of 72 [56.5, 78.0] and poor symptom control, as defined by an Asthma Control Questionnaire (ACQ6) result of 2.4 [1.28, 3.2]. A high perceived barriers to exercise score was significantly correlated with increased asthma symptoms (*r* = 0.452, *p* < 0.0001), anxiety (*r* = 0.375, *p* = 0.005) and depression (*r* = 0.363, *p* = 0.008), poor quality of life (*r* = 0.345, *p* = 0.015) and number of rescue oral steroid courses in the past 12 months (*r* = 0.257, *p* = 0.048). Lung function, blood eosinophil count, FeNO, Njimegen and SNOT22 scores, BMI and hospitalisations in the previous year were not related to exercise perceptions.

**Conclusion:**

In difficult asthma, perceived barriers to exercise are related to symptom burden and psychological morbidity. Therefore, exercise interventions combined with psychological input such as CBT to restructure thought processes around these perceived barriers may be useful in facilitating adoption of exercise.

## Background

Exercise is recommended in national and international guidelines for asthma management [1–3] and appears to have beneficial effects on symptom control, inflammation and lung function in patients with sub-optimally controlled asthma [4]. Despite this, physical activity levels in patients with severe asthma have been demonstrated to be impaired [5]. Patients with difficult and severe asthma comprise only 5–10% of all patients with asthma [6, 7]. However, they are disproportionately more likely to demonstrate poorly controlled symptoms and inflammation on optimised treatment regimens. This drives a significant proportion of healthcare costs, reported to consist of near to 50% of total asthma therapy costs [6, 8]. In related disease areas, exercise interventions are being offered at scale using novel technologies such as healthcare apps to increase patient centred management in COPD which could be harnessed for prevalent diseases such as difficult asthma [9–12]. Understanding of the barriers to exercise is crucial in increasing activity in patients with difficult asthma, and in implementing a successful exercise training programme to improve their health outcomes [13, 14].

In the general population, reasons for physical inactivity are due to a combination of insufficient leisure time and increased mechanization of occupational and domestic activities [15]. In patients with asthma there may be additional disease related barriers to exercise such as fear of provoking respiratory symptoms and exacerbation, and misinterpretation of physiological shortness of breath in response to increased aerobic activity. Understanding these may facilitate design of exercise interventions.

Alongside patients with severe asthma, patients with relatively mild disease have been shown to avoid physical activity because they are concerned about triggering symptoms [16]. However, asthma severity, as assessed by FEV_1_ and methacholine challenge, were not predictive of VO2 (maximal oxygen uptake) peak as a marker of aerobic fitness, and the relationship between asthma severity and VO2 max has been detailed in athletic individuals previously. These findings suggest that disease severity does not determine fitness in asthma patients who manage to overcome perceived barriers to exercise and undertake regular physical activity [16, 17]. Relatively few studies have investigated the barriers and facilitators to exercise and physical activity in asthma. However, those which have focus predominantly on adolescents. This is partly because asthma tends to affect younger populations in childhood and adolescence at a time when they should be establishing healthy lifestyles. This is therefore a critical point for intervention to encourage long-term adoption of physical activity [18]. Whilst qualitative studies suggest healthy participants and asthma patients consider that exercise as beneficial [19], a study of elementary school teachers demonstrated few were aware that students with asthma need not avoid exercise [20]. Other barriers have also been identified that prevent this group of patients engaging with physical activity. For example, lack of time is more likely to be reported as a barrier in younger patients [19]. Fear of exacerbating symptoms is also a common theme amongst adolescents [21] and adults [19], with patients with more severe disease more likely to view exercise as detrimental. Intensity of physical activity undertaken by asthma patients has been shown to be positively correlated with peak expiratory flow [22]. Although causation could not be determined in this cross-sectional study, it raises the question as to whether those with less severe disease are able to undertake more activity or whether those who undertake more activity are able to modulate their disease burden, as supported by findings in a recent review [23]. Obesity and musculoskeletal problems are conditions that are common in asthma and exacerbated by oral steroid therapy. These are also reasons for this patient population not exercising, as were extreme weather conditions [19]. Facilitators included the desire to be healthy and encouragement from a motivated companion or physician. Lifestyle activities have been shown to be more acceptable to patients as a way to increase their physical activity levels [19]. In terms of intrinsic characteristics, patients with less asthma knowledge, lower self-efficacy and more negative attitudes towards asthma were more likely to view exercise negatively [19]. Similar themes were noted in a group of middle aged African American women with poorly controlled asthma who participated in focus groups to determine barriers to walking. Domains identified in this group included limited physical capability, lack of knowledge, lack of self-monitoring skills, lack of areas to walk, lack of social support and beliefs about consequences and capability [24].

In this paper we present the perceived barriers to exercise in patients with difficult asthma in a group recruited from the Wessex Asthma Cohort of Difficult Asthma (WATCH). Furthermore, we assess their relationships to aspects of asthma severity and control.

## Methods

### WATCH Data Collection

WATCH is a longitudinal clinical cohort of patients with Difficult Asthma (*n* = 501) based at University Hospitals Southampton NHS Foundation Trust (UHSFT), Southampton, United Kingdom (UK). All patients managed with British Thoracic Society Step “high dose therapies” and/or “continuous or frequent use of oral steroids” [1] in the Adult or Transitional Regional Asthma Clinic at UHSFT were invited to participate. Briefly, research data capture was aligned with the extensive clinical characterisation required of a commissioned National Health Service (NHS) Specialist Centre for Severe Asthma [25]. Data acquisition at enrolment included detailed clinical, health and disease-related questionnaires (Asthma Control Questionnaire (ACQ6), St George’s Respiratory Questionnaire (SGRQ) and EQ-5D-5 L, Njimegen questionnaire for dysfunctional breathing, Sinonasal Outcome Test (SNOT22) for sinonasal symptom burden and Hospital Anxiety and Depression score (HADS) for anxiety and depression), anthropometry, allergy skin prick testing (SPT), lung function testing, radiological imaging (in a subset of those who were clinically indicated) and collection of biological samples (blood, and urine). Brief longitudinal updates of data were obtained annually. A detailed outline of study protocol and methodology has previously been published [25]. The Exercise Therapy Burden Questionnaire (ETBQ) has been validated in French and Spanish for the assessment of barriers to physical activity in chronic illness and consists of 10 questions graded from 0 to 10; a higher score indicates higher perceived barriers to exercise [10, 26, 27] (see supplement for questionnaire). Ninety patients were approached to complete an ETBQ, either as part of their WATCH enrolment, or whilst they were attending a routine clinic follow-up visit between January 2019 and February 2020. Those patients who did not attend clinic during this time or were not due a WATCH follow up visit during this period may not have been approached. A total of 62 patients fully completed the questionnaire. Data were then extracted for the clinical correlates which most temporally associated with the ETBQ completion. The primary outcome was to identify whether a higher asthma disease burden was related to greater perceived barriers to exercise. Secondary outcomes focused on relationship between barriers to exercise and specific areas of asthma disease burden.

### Data Analysis

Statistical analysis was performed using SPSS 24 (NY, USA), and GraphPad Prism 8 (La Jolla, California, USA). Non-parametric tests were used due to some of the data being non-normally distributed. Quantitative variables are presented as median and inter-quartile range (IQR). Mann-Whitney and Fisher’s exact tests were used to compare the WATCH cohort as a whole with the ETBQ cohort. Results for these variables were compared using an independent samples Mann Whitney test, Kruskall Wallis and Independent Samples Median tests were used to look for differences between groups. Associations between variables were tested using a Spearman’s Rho test. A *p* value of < 0.05 was considered statistically significant.

## Results

### Demographic data

The sub cohort of patients who completed the ETBQ were comparable in most core characteristics to the wider WATCH cohort (Table 1). The only significant differences between the ETBQ group and the overall WATCH cohort were that the cohort as a whole had a higher mean [95% confidence interval] use of rescue oral corticosteroids (OCS) (3.60 [3.24, 3.96] vs 1.93 [1.24, 2.62], *p* < 0.0001), a higher rate of hospitalisation in the previous 12 months (0.76 [0.59, 0.93] vs 0.24 [0.01, 0.47], *p* = 0.0025), a lower FeNO (31.1 [27.5, 34.8] vs 48.55 [16.5, 80.6], *p* = 0.03) and a higher HADS-D score (5.4 [5.0, 5.8] vs 4 [0.1, 5.0], *p* = 0.04). Biologic use was higher in the ETBQ group than the WATCH cohort overall (39% vs 18%, *p* = 0.0016), demographic and disease-related characteristics of the ETBQ group are given in Table 1.

**Table 1:** Demographic and disease related data

	*WATCH Cohort as a whole (n)*	Median [IQR]	*N (%)*	*EBTQ Cohort Baseline Data (n)*	Median [IQR]	*N (%)*	*P value*
*Demographics*							
Female	501		65.3%	62		69.4%	ns
Age at Study Enrolment (years)	501	52 [38.5, 63.0]		62	53.5 [35.75, 65.25]		ns
Age at asthma diagnosis	479	19 [4, 40]		62	23 [3.0, 40.35]		ns
BMI	495	29.7 [25.6, 35.3]		60	29.25 [25.5, 36.23]		ns
Obese	*495*		48.3%	62		48.3%	ns
Current or Ex Smokers	*500*		47.6%	62		31.1%	ns
*Co-Morbidities*							
Rhinitis	*446*		67.5%	62		58.1%	ns
Eczema	*495*		26.1%	62		25.8%	ns
Bronchiectasis	*493*		6.9%	62		16.1%	ns
GORD	*495*		14.1%	61		50%	ns
Depression	*486*		64.8%	62		17.7%	ns
Anxiety	*454*		36.8%	62		19.4%	ns
Dysfunctional Breathing	*451*		48.7%	61		41%	ns
Intermittent Laryngeal Dysfunction	*476*		14.5%	59		10.2%	ns
Sulphite Sensitivity	*447*		7.7%	62		4.8%	ns
Salicylate Sensitivity	*493*		25.1%	62		21%	ns
Sleep Apnoea			7.2%	62		6.5%	ns
*Healthcare Utilisation*							
≥ 1 Asthma Related ICU Visits ever	*500*		28.2%	60		1.7%	ns
≥ 1 Asthma Hospital Admission (last 12 months)	*497*		29.0%	62		11.3%	*P* = 0.0025
≥ 3 Rescue Oral Corticosteroids (last 12 months)	*448*		43.6%	60		31.7%	ns
Maintenance oral corticosteroids steroids	*479*		29.9%				*P* < .0001
Biological treatment in last 12 months	*495*		17.6%			39%	*P* = .0016
*Blood Test Results*							
Eosinophil Count					0.2 [0.1, 0.4]		ns
*Lung Function Test Results*							ns
FeNO50 (ppb)	329	19.7 [10.0, 38.7]		62	22 [14, 45.5]		*P* = .03
Post BD FEV1 (%)	341	75 [59.3, 92.1]		57	73.4 [59.5, 86.6]		ns
Post BD FEV1/FVC (ratio)	340	68 [58, 78]		57	72 [56.5, 78]		ns
*Skin Prick Tests*							
Positive to any Aeroallergen	*391*		68.0%	52		75%	ns
Positive to Aspergillus	*355*		15.8%	47		17%	ns
*Questionnaires*							
ACQ6 Score	467	2.5 [1.5, 3.5]		62	2.4 [1.28, 3.2]		ns
Epworth Score	424	8 [4, 12.75]		55	8 [3, 11]		ns
HADS Total Score	418	10.5 [6, 18]		53	8 [4.0, 15.5]		ns
HADS A Score	425	6 [3, 10]		55	5 [3, 9]		ns
HADS D Score	426	4 [2, 8]		53	3 [1, 6]		*P* = .04
Hull Cough Score	378	25 [14, 36]		48	30 [14.25, 41.75]		ns
Nijmegen Score	373	21 [12, 31]		47	21 [13, 26]		ns
SNOT22 Score	324	31.5 [20, 50]		40	36.5 [23.25, 48.75]		ns
EQ_5D_5L Index value	170	0.72 [0.53, 0.83]		62	0.72 [0.54, 1.00]		ns
SGRQ Total Score	381	51.1 [35.25, 67.34]		49	59.6 [37.1, 63.4]		ns
SGRQ Symptoms Score	411	67.73 [50.72, 81.31]		53	68 [53, 81.7]		ns
SGRQ Activity Score	389	66.1 [43.7, 85.7]		50	66.2 [41.8, 73.8]		ns
SGRQ Impacts Score	396	38.71 [22.76, 55.74]		52	36 [25.4, 54.1]		ns

### Barriers to exercise results

There were no significant differences between those patients who completed the questionnaire and the overall group of patients who were approached to complete the questionnaire in terms of median BMI (30.00 vs 29.65, *p* = 0.9), FEV1 (73.43 vs 73.43, *p* = 0.83, ACQ 6 score (2.4 vs 2.5, *p* = 0.45), OCS courses (1 vs 1, *p* = 0.77), hospitalisations (0 vs 0, *p* = 0.24), blood eosinophils (0.2 vs 0.2, *p* = 0.7) or FeNO (21 vs 20, *p* = 0.55). Verbal feedback from patients who were approached but did not complete the questionnaire suggested reasons for not doing so which included time required to complete, and uncertainty regarding the relevance of the questionnaire if they had not been specifically prescribed an activity. 49 (79%) of the patients who fully completed the questionnaire took part in some focussed physical activity, with 18 (29%) stating that they played sports, 11 (17.7%) attending physiotherapy sessions and 20 (32.3%) undertaking a home-based exercise programme. There was a median (IQR) total score of 25.5 [11.25, 42.75] out of a possible total score of 100.

**Fig. 1:**
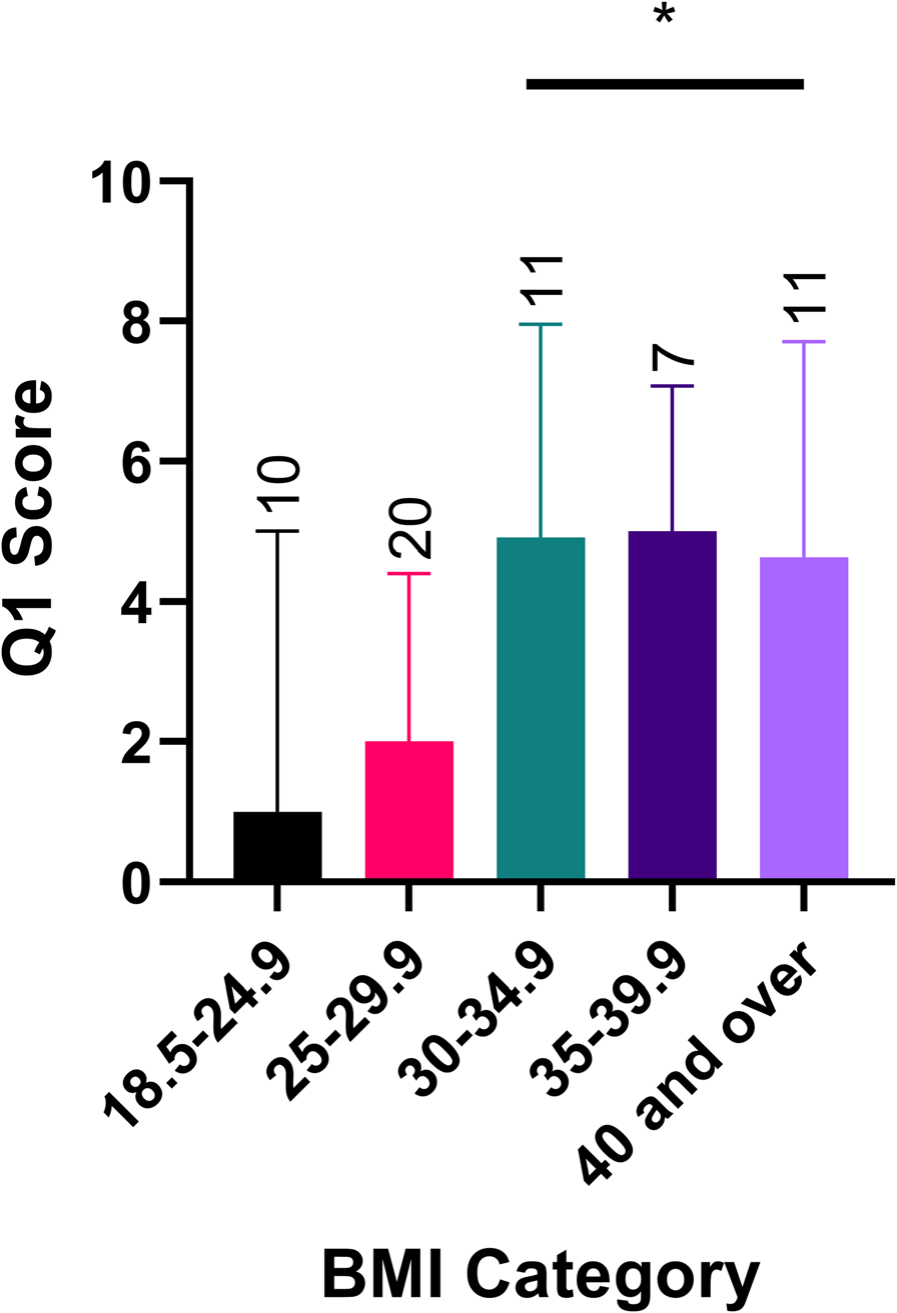
Q1 (The exercise causes me pain): results for comparison using Kruskal Wallis Test to compare ETBQ scores for question 1 when grouped by BMI category (mdn and IQR), with significantly higher scores noticed in those overweight (*p* = 0.017)

Median [range] results for the specific questions within the ETBQ are shown in Table 2. Motivation (3 [0–10]), pain or discomfort (4 [0–10]), fatigue (5 [0–10)) and being reminded of their asthma (5 [0–10]) were the most limiting factors to exercise programmes within this group. There were no significant differences for individual questions or overall score when grouped by gender (*p* = 1). There were no significant differences in total scores when grouped across age range (*p* = 0.479) or body mass index (*p* = 0.671). However, when the individual question scores were analysed by body mass index, there was a significant difference in scores for question 1 (The exercise causes me pain) for patients when group by BMI (*p* = 0.017) (Fig. 1). However, post-hoc pairwise comparisons were not significant once adjusted for multiple testing.

**Table 2:** ETBQ question results for total cohort

Question	*Median*	Range
Q1 (Pain or discomfort)	4	0–10
Q2 (Fatigue)	5	0–10
Q3 (Boredom)	1	0–10
Q4 (Too Difficult)	2	0–9
Q5 (Wastes Time)	0	0–9
Q6 (Reminds of Condition)	5	0–10
Q7 (Lacks Support)	0	0–10
Q8 (Lacks Motivation)	3	0–10
Q9 (Inappropriate)	0	0–9
Q10 (Not Efficient)	0.5	0–10

*Median (and min-max) results for each of the ten questions comprising the ETBQ for the total cohort are shown (n = 62)*

**Fig. 2:**
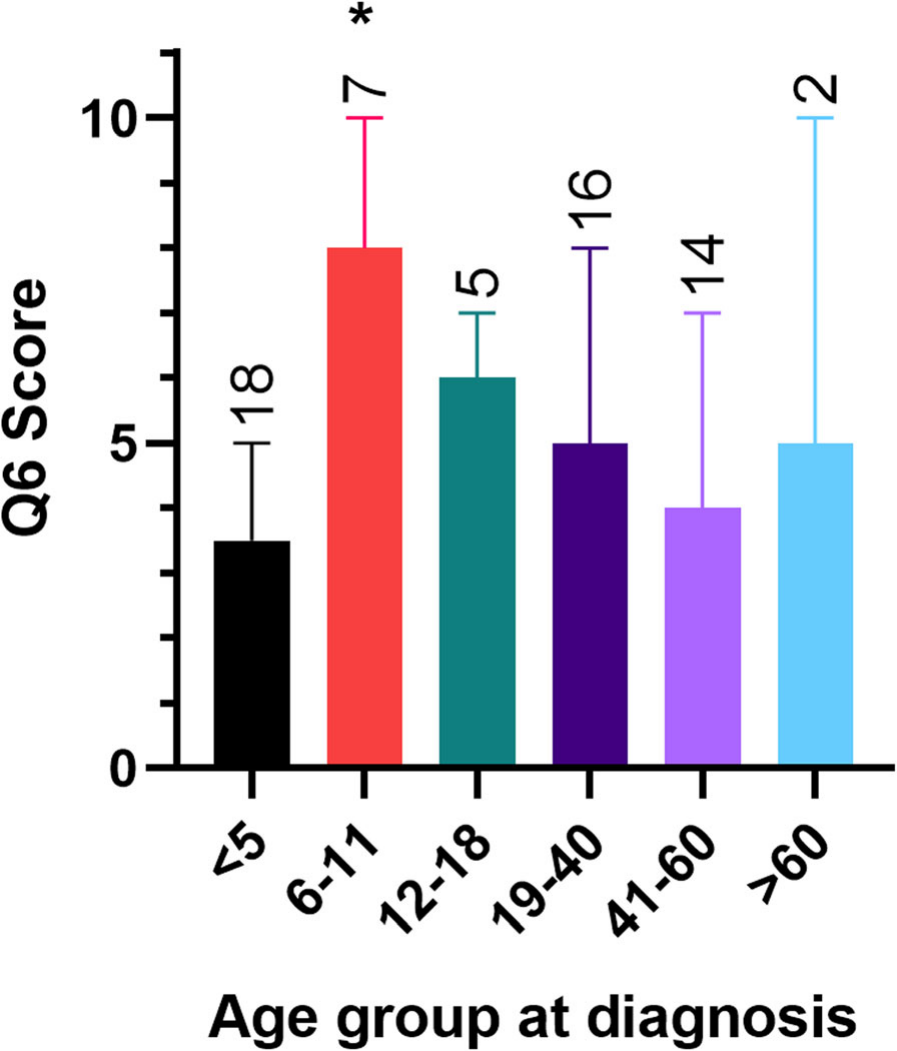
Q6 (Exercising reminds me of my condition)-: Independent samples Median Test results for comparison of ETBQ scores for question 6 when grouped by age at diagnosis (mdn and IQR), with significant differences in the age 6–11 group (*p* = 0.03)

When individual question scores were analysed for differences across the age range, there were significant differences in scores for question 6 (exercising reminds me of my condition), with those in the diagnosed at the age of 6–11 group scoring significantly higher than those in the 5 years and under group (*p* < 0.05) (Fig. 2).

### Relationships between ETBQ score and asthma related assessments

**Fig. 3:**
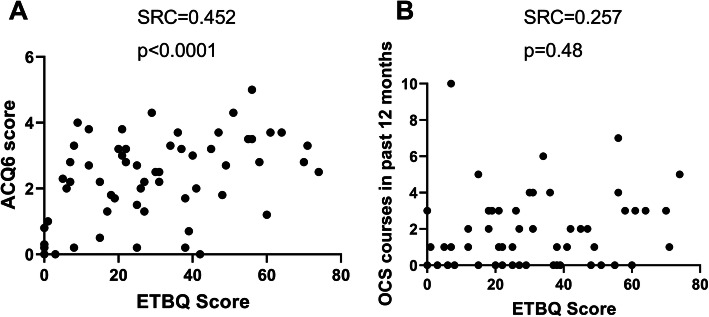
Correlation between symptom scores (ACQ6, Fig. 4a) and rescue OCS (Fig. 4b) as assessed by Spearman Rank Correlation with r and *p* values

**Fig. 4:**
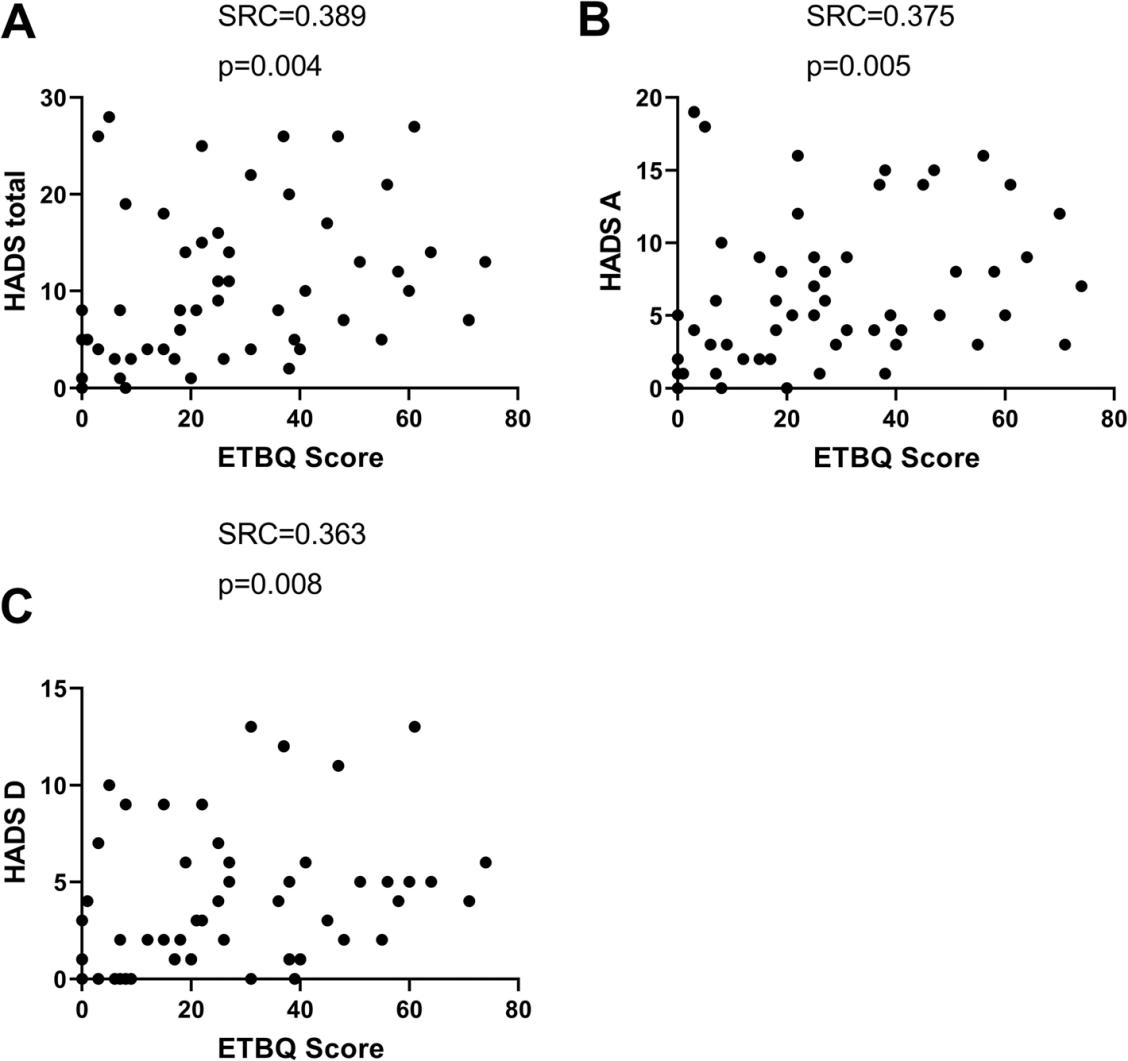
ETBQ and psychological comorbidity for anxiety and depression (HADS total, Fig. 5a), anxiety (HADSA, Fig. 5b), depression (HADSD, Fig. 5c), as assessed by Spearman Rank Correlation with r and *p* values

**Fig. 5:**
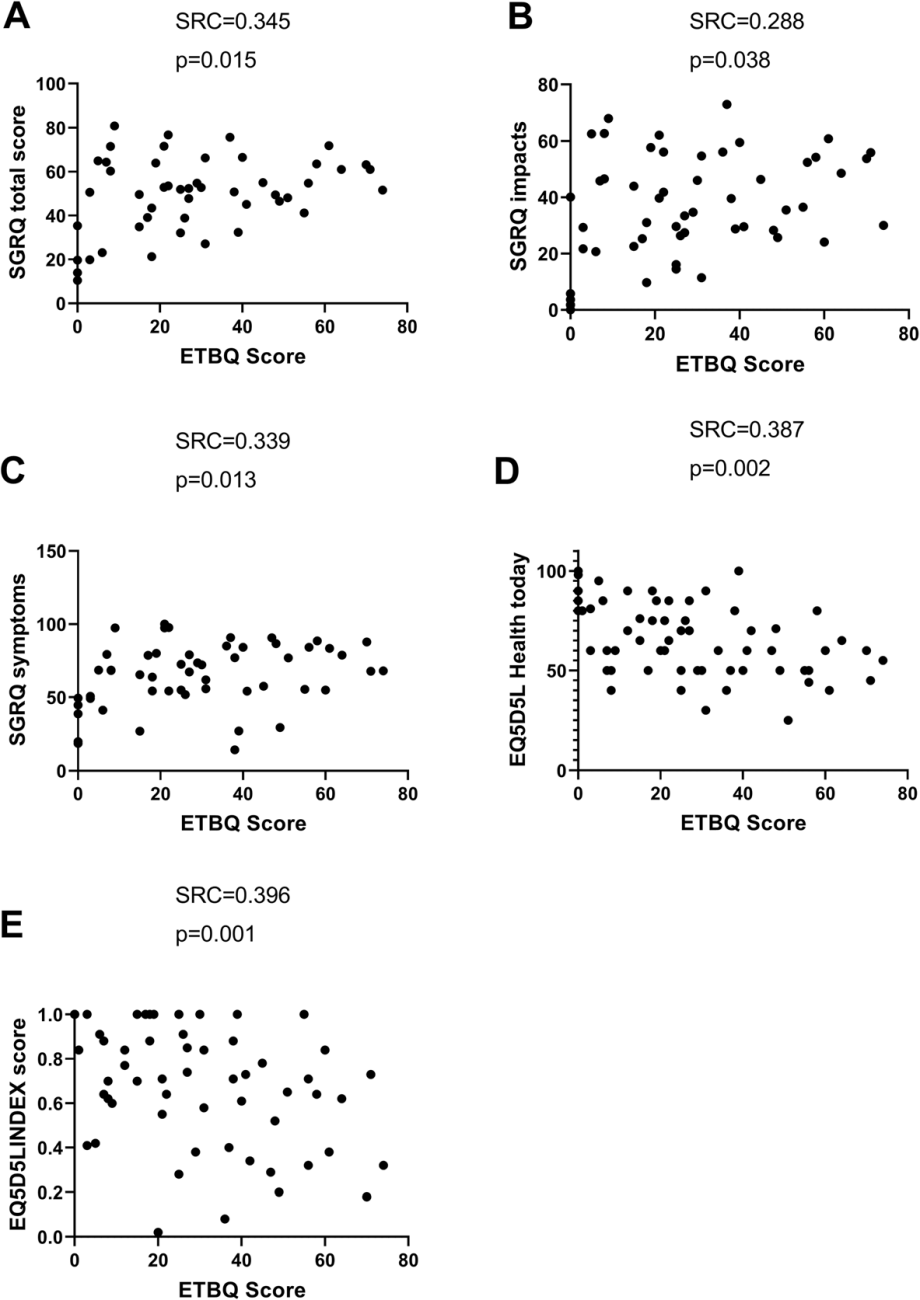
ETBQ and Quality of Life Scores for SGRQ total (6A), impacts (6B) and symptoms (6C), and EQ-5D5L heath today 6D, and EQ-5D-5 L Index (6E as assessed by Spearman correlation, with r and *p* value

We then looked at relationships between a high total ETBQ score and markers of asthma severity and symptom burden. High perceived barriers to exercise scores were significantly correlated with increased asthma symptoms, as measured by the Asthma Control Questionnaire (ACQ6) (*r* = 0.452, *p* = <.0001), and number of rescue OCS uses in the past 12 months (*r* = 0.257, *p* = 0.048) (Fig. 3). Psychological co-morbidities in the form of anxiety and depression were assessed by the Hospital Anxiety and Depression Score (HADS). There was significant correlation between high perceived barriers to exercise therapy and high HADS scores, both for anxiety (*r* = 0.363, *p* = 0.008) and depression (*r* = 0.375, *p* = 0.002), independently and as a total score (*R* = 0.389, *P* = 0.004) (Fig. 4). Low perceived quality of life scores were assessed by the EQ-5D-5 L and the St George’s Respiratory Questionnaire (SGRQ) and correlated with a higher perceived barriers to exercise (Fig. 5). Lung function (pre BD FEV1; *r* = − 0.087, *p* = 0.522), eosinophil count (*r* = 0.154, *p* = 0.235), FeNO, Nijmegen (*r* = 0.213, *p* = 0.151) and SNOT22 scores (*r* = − 0.078, *p* = 0.151), BMI (*r* = 0.180, *p* = 0.168) and hospitalisations (*r* = − 0.78, *p* = 0.548) in the previous year were not significantly correlated with ETBQ score. There were no statistically significant differences in total ETBQ score or individual question scores when participants were divided by biologic use in the last 12 months. No significant differences in lung function results, eosinophil counts and FeNO were seen for those on and not on biological treatments.

## Discussion

### Perceived barriers to exercise in patients

We assessed the perceived barriers to exercise in patients with difficult asthma under the care of a tertiary clinical service to create a better real-life understanding of relevant limiting factors. To the best of our knowledge, this is the first study to explore this in patients with difficult asthma. Although differences were seen in OCS and rates of hospitalisation, our patient group was generally comparable to the WATCH Cohort as a whole, and representative of a typical group of patients with difficult asthma. Patient perceptions of barriers to exercise in difficult asthma were high. The median score within our cohort were comparable to those found in patients with cardiovascular disease and much higher than those seen in patients with cancer [28]. The distribution of scores was wide throughout the cohort, suggesting that perceived barriers to exercise are patient specific, possibly reflecting the heterogeneity of difficult asthma. Identification of values of low, medium and high scores for the ETBQ have not been identified, but may be useful to identify in the future.

There did not appear to be significant differences between sex of the patient and perceived barriers to exercise. This contrasts with a previous study which investigated perceived barriers to exercise in a cohort of university students with disabilities. This study demonstrated that the most significant barriers to exercise were interpersonal in nature and that females were more likely to experience higher interpersonal barriers [29]. It may be that within our difficult asthma patient group, the disease related barriers to exercise were great enough to balance out any sex specific barriers.

The lack of correlation with frequency of hospitalisations is interesting, suggesting that exacerbations on a background of reasonable day to day control may impact less on perceptions of barriers to exercise than a constant level of poor control with few exacerbations. This may be of relevance to prescribing criteria for biological treatments, which, at present focus on exacerbation frequency [30].

### BMI impacts perceived barriers to exercise

There was a significant difference for Q1 score in BMI categories, as identified by the overall Kruskall Wallis test. The post-hoc pairwise comparisons were not significant. However, this was probably due to a lack of statistical power due to small group sizes. This effect of BMI on perceived barriers to exercise is noteworthy, but not a wholly unexpected finding as differences between BMI and barriers to physical activity have previously been demonstrated in young Australian males [31]. Patients with asthma who are obese have a greater symptom burden and lose more days to illness than non-obese asthma patients [32]. This population are more likely to benefit from exercise interventions to address both obesity and asthma driven inflammation [33, 34]. It is therefore important to adapt current exercise interventions to make them more accessible to this group of patients, potentially with classes available for this group of patients specifically to help alleviate concerns around others’ perceptions, and psychological and dietician support alongside this. Understanding that perceived barriers to exercise differ for obese patients with asthma is the essential first step in doing this. Further work investigating the specific causes of pain in these patients is now important. The lack of correlation between BMI and overall perceived barriers to exercise is interesting. It may be that the majority of the perceived barriers to exercise in obese patients is related to pain, but overall the perception of barriers to exercise was not increased by a BMI. BMI is a gross measure of obesity, and noted to be overestimated in those with high muscle mass. This may add further ambiguity to results, and investigation of bioimpedence data would perhaps give further clarification.

### Differentially perceived barriers to exercise dependent on age of diagnosis

In this present study, significantly different perceptions on the effect of asthma to barriers to exercise were demonstrated between groups based on age of diagnosis. Those whose disease started between the ages of 6–11 were more likely to see their disease as a barrier to exercise than those diagnosed under 5 years old. This appears to be a key stage for engagement in sport in later life, with a report from The Women in Sport Research group showing that if children start to drop out of sporting activities at this age then they tend not to re-engage as adults [35]. Comparatively, exercise levels in children at age 7 are not reduced in those with a diagnosis of asthma [36]. It may be that diagnosis at this age compounds the effects of this transition point. Diagnosis at this age may result in a higher dropout rate from physical activity which continues into adulthood. This could partly explain some of the lower levels of activity seen in patients with asthma compared to the general population. Targeted interventions in this age group may go some way to ameliorating this effect [5].

### Perceived symptom burden impacts perceived barriers to exercise

A high perceived symptom burden as assessed through the symptom scores (ACQ6) and number of rescue courses of OCS were found to significantly correlate with an increased perceived barrier to exercise. Correlations between a perceived high barrier to exercise therapy and disease specific assessments are reflective of the literature. Those with more severe disease have previously been shown to view exercise as more likely to be detrimental [19]. Both these measures of symptom burden are partially subjective. ACQ scores reflect patient interpretation of their symptoms over the preceding week. Furthermore, rescue courses of OCS are often started by patients as part of a rescue pack on the basis of deteriorating symptoms. However, in this present research, objective asthma specific markers of severity such as lung function and markers of Type 2 high disease did not correlate with perceived barriers to exercise. Similarly, a cross-sectional analysis of physical activity in the UK millennium cohort demonstrated that activity levels in children with asthma were not affected by the severity of their disease [36]. This is a clinically relevant finding which suggests that severity of disease is not necessarily a barrier to exercise. This has been supported by our pilot work [37], and that of others [33], investigating exercise intervention in asthma patients. This suggests that high levels of biological disease are not necessarily a barrier to adoption of exercise for some patients. This data is of use for reassuring both patients and clinicians that exercise intervention is safe in asthma regardless of disease severity.

Psychological co-morbidity in the form of a high HADS Anxiety, depression and total scores also correlated significantly with a higher perceived barriers to exercise score. A meta-analysis has previously identified low mood and stress as two of the most significant barriers to exercise in mental illness [38]. However, exercise has also been demonstrated to improve mood associated with reduction in depression-associated inflammation in COPD [39] and in health [40]. A similar pattern has been seen with QoL where exercise specific self-efficacy has been shown to correlate with health related QoL in COPD [41]. Therefore, our results which show that a higher barrier to exercise correlates with a lower QoL score are not unexpected. Exercise is, however, known to improve health related QoL in asthma [4, 23] and therefore interventions to address this paradox need investigating.

### Challenges and considerations

There are limitations to this study. Firstly, the strength of correlations throughout were low-moderate; this was likely a result of the numbers who completed the questionnaire. Secondly, a questionnaire format will not provide as detailed or accurate information as a qualitative interview format. However, there are advantages to a questionnaire format, in that participants may be more honest with regards to barriers to exercise than they would be with a face-face interviewer. Furthermore, a questionnaire format reduces time demands on patients and clinical staff both in a research and clinical context, whilst still providing noteworthy findings, which could then potentially be expanded on in a qualitative way. The ETBQ was the most specific questionnaire available at the time of conception of this study to address the question of perceived barriers to exercise within the context of a chronic disease, thus this questionnaire was chosen to be used.

With regards to other limitations, asthma symptoms can fluctuate and the clinical data was not necessarily collected at the same time as the ETBQ. However, the clinical data which most closely aligned temporally with the ETBQ data was extracted from the database to reduce any inaccuracies. Also, questionnaires were completed at different stages of enrolment in the WATCH study; some at baseline, and others at follow up visits. Similarly, perceived patient barriers to exercise may change depending on the day of the exercise, and this may not be captured by a single time point questionnaire. There were a few significant differences between the WATCH cohort and the ETBQ sub-cohort, including number of rescue courses of OCS in the last 12 months, which was higher in the WATCH cohort as a whole. This may partly explain the only borderline significance of the correlation between ETBQ total score and OCS rescue courses. Besides this, the ETBQ cohort was representative of the wider WATCH population and there was no difference between those who completed the questionnaire compared to those who did not, suggesting this was not a bias to taking part in the ETBQ study. The ETBQ focuses on a prescribed activity and yet some patients within the cohort were not prescribed any activity. If this were the case, then they were asked to complete the questionnaire from the perspective of what prevents them from exercising rather than the burden of any prescribed exercise. However, misunderstanding of this may explain why only 62 of the 90 participants invited to complete the ETBQ fully completed the questionnaire. With any self-reported questionnaire-based research, there is always the concern of responder bias. However, patients were asked to complete the questionnaire regardless of whether they undertook regular exercise. This removed any expectation that they should be taking part in exercise.

## Conclusion

Patient perceived barriers to exercise are more related to symptom burden and psychological morbidity than to specific disease severity indicators. Therefore, exercise interventions combined with psychological input such as CBT to restructure thought processes around these perceived barriers may be useful in facilitating adoption of exercise.

## Supplementary information

**Additional file 1.** Exercise Therapy Burden Questionnaire.

## Data Availability

All data generated or analysed during this study are included in this published article [and its supplementary information files].

## Abbreviations

ACQ: Asthma Control Questionnaire
BD: Bronchodilator
BMI: Body Mass Index
ETBQ: Exercise Therapy Burden Questionnaire
FeNO: Fractional Exhaled Nitrogen Oxide
FEV1: Forced Expiratory Volume in 1 s
FVC: Forced Vital Capacity
HADS: Hospital Anxiety and Depression Score
ICU: Intensive Care Unit
OCS: Oral corticosteroids
SGRQ: St George’s Respiratory Questionnaire
SNOT: 22 Sinonasal Outcome Test
