# Supplementary material for: Patient perceived barriers to exercise and their clinical associations in difficult asthma
Source: Asthma Res Pract. 2020 Jun 9;6:5. doi: 10.1186/s40733-020-00058-6 (PMC7285728; doi:10.1186/s40733-020-00058-6)
Supplement: Supplementary file 1 — Additional file 1. Exercise Therapy Burden Questionnaire. [file 40733_2020_58_MOESM1_ESM.docx]

**Supplement:**

***Exercise Therapy Burden Questionnaire***

Your physician has asked you to play sports, to exercise on your own or to attend sessions with a physiotherapist to treat your condition.

Here are some of the statements we have heard from other patients about difficulties or constraints in following the recommendations or prescriptions they were given about physical exercise in treating their condition. We call "burden" all of these difficulties and constraints that can hinder you in carrying out your exercises.

For each statement, please select a number from 0 to 10 to indicate how some of the difficulties or constraints expressed by these patients also affect you in performing your exercises. The number you choose will express how much the stated proposition is a difficulty or a constraint to your physical exercises.

Please tell us first what is the main prescribed or recommended physical activity you are doing:

☐ *Sports* ☐ *Physiotherapy* ☐ *Home based exercise program*

1. The exercises cause me pain :

| Not at all | ☐ ☐ ☐ ☐ ☐ ☐ ☐ ☐ ☐ ☐ ☐  0 1 2 3 4 5 6 7 8 9 10 | All the time |
| --- | --- | --- |

1. The exercises cause me fatigue :

| Not at all | ☐ ☐ ☐ ☐ ☐ ☐ ☐ ☐ ☐ ☐ ☐  0 1 2 3 4 5 6 7 8 9 10 | All the time |
| --- | --- | --- |

1. I get bored when I exercise (too much repetition, not enough fun) :

| Not at all | ☐ ☐ ☐ ☐ ☐ ☐ ☐ ☐ ☐ ☐ ☐  0 1 2 3 4 5 6 7 8 9 10 | All the time |
| --- | --- | --- |

1. The exercises to achieve in my program are too difficult:

| Not at all | ☐ ☐ ☐ ☐ ☐ ☐ ☐ ☐ ☐ ☐ ☐  0 1 2 3 4 5 6 7 8 9 10 | All the time |
| --- | --- | --- |

1. I waste too much time exercising:

| Not at all | ☐ ☐ ☐ ☐ ☐ ☐ ☐ ☐ ☐ ☐ ☐  0 1 2 3 4 5 6 7 8 9 10 | All the time |
| --- | --- | --- |

1. Exercising reminds me of my condition:

| Not at all | ☐ ☐ ☐ ☐ ☐ ☐ ☐ ☐ ☐ ☐ ☐  0 1 2 3 4 5 6 7 8 9 10 | All the time |
| --- | --- | --- |

1. I lack support to exercise:

| Not at all | ☐ ☐ ☐ ☐ ☐ ☐ ☐ ☐ ☐ ☐ ☐  0 1 2 3 4 5 6 7 8 9 10 | All the time |
| --- | --- | --- |

1. I lack motivation to exercise:

| Not at all | ☐ ☐ ☐ ☐ ☐ ☐ ☐ ☐ ☐ ☐ ☐  0 1 2 3 4 5 6 7 8 9 10 | All the time |
| --- | --- | --- |

1. The exercises that I am asked to do are not adapted to my physical activity objectives

| Not at all | ☐ ☐ ☐ ☐ ☐ ☐ ☐ ☐ ☐ ☐ ☐  0 1 2 3 4 5 6 7 8 9 10 | All the time |
| --- | --- | --- |

1. I feel that exercising is not efficient in my case:

| Not at all | ☐ ☐ ☐ ☐ ☐ ☐ ☐ ☐ ☐ ☐ ☐  0 1 2 3 4 5 6 7 8 9 10 | All the time |
| --- | --- | --- |
